# Machine learning in obsessive-compulsive disorder medications

**DOI:** 10.1016/j.heliyon.2024.e40136

**Published:** 2024-11-05

**Authors:** Mahdiyeh Khazaneha, Behnaz Bakhshinejad, Mitra Mehrabani, Abdolreza Sabahi, Mohammad Khaksari, Mehdi Shafiee, Mohsen Nakhaie, Mohammad Rezaei Zadeh Rukerd, Abdollah Jafarzadeh, Mehrzad Mehrbani

**Affiliations:** a Neurology Research Center, Kerman University of Medical Sciences, Kerman, Iran; b Physiology Research Center, Institute of Neuropharmacology, Kerman University of Medical Sciences, Kerman, Iran; c Department of Traditional Medicine, Faculty of Persian Medicine, Kerman University of Medical Sciences, Kerman, Iran; d Herbal and Traditional Medicines Research Center, Kerman University of Medical Sciences, Kerman, Iran; e Neuroscience Research Center, Neuropharmacology Research Institute, and Department of Psychiatry, School of Medicine, Shahid Beheshti Hospital, Kerman University of Medical Sciences, Kerman, Iran; f Department of Electrical Engineering, National University of Skills (NUS), Tehran, Iran; g Gastroenterology and Hepatology Research Center, Institute of Basic and Clinical Physiology Sciences, Kerman University of Medical Sciences, Kerman, Iran; h Department of Immunology, School of Medicine, Kerman University of Medical Sciences, Kerman, Iran

**Keywords:** Machine learning, Decision tree, Obsessive-compulsive disorder, OCD, Clomipramine, Duloxetine

## Abstract

Obsessive-compulsive disorder (OCD) is the fourth most common psychiatric disorder with a significant morbidity rate. Despite various treatment modalities and medications, some patients show no definitive response. The aim of this study is to classify the medications of OCD with machine learning (ML) methods and to compare the classification performances of the decision tree (DT), chi-square automatic interaction detection (CHAID) algorithm, and linear model in ML methods. This research is a descriptive analytical study based on co-word and artificial intelligence methods. The DT models were created with a target (total weight link strength). For hyperparameter optimization, the Gini index was used as the weight total link strength. The performance of the DT model was evaluated based on the prediction model. A total of 116 drugs were extracted from 6574 articles based on co-word analysis, and 56 drugs were classified as the DT's root. These drugs were categorized into six groups in the EWKM diagram. The DT was constructed using the weight.total.link index, with 7 items in Label 3 and 42 items in Label 5 serving as DT leaves. The ML analysis provided valuable insights into the efficacy of various medications such as clomipramine, duloxetine, and pindolol, as well as supplements such as folate, in the treatment of OCD. Treating concomitant diseases, namely hypothyroidism and streptococcal infection could improve the efficacy of treatment.

## 1 Introduction

According to the World Health Organization (WHO), obsessive-compulsive disorder (OCD) is one of the ten diseases that cause disability and reduce quality of life worldwide [1]. This disorder manifests with a variety of symptoms, such as disturbing thoughts, obsessive habits, mental preoccupations, and practical obsessions [2]. The American Psychiatric Association's Fifth Diagnostic and Statistical Manual of Mental Disorders (DSM-V) (2013) defines OCD as "a series of intrusive thoughts or images (obsessions) that cause severe distress and anxiety in a person". It is the fourth most common psychiatric disorder, which has a chronic and often debilitating pattern. This disease affects men and women equally, with a prevalence ranging from 1.3 % to 3.5 % [3].

OCD typically manifests at the age of 20 [4]. This disorder is caused by a combination of biological, genetic, psychological, and behavioral factors [5]. Pharmacological and behavioral treatments are the main forms of therapy for this disorder. Selective serotonin reuptake inhibitors (SSRIs) and clomipramine, a tricyclic antidepressant, are the most commonly used medications [[5], [6], [7]]. However, despite various treatment modalities and medications, some patients show no definitive response [8,9]. One of the most important steps in the drug discovery process is identifying drug interactions and their effects, which can be carried out using a variety of approaches, including machine learning (ML) [10,11].

ML is a prediction method based on large datasets and flexible model architecture. Its tasks are classified as supervised, unsupervised, or semi-supervised learning. Because supervised regression is applied to predict problem solutions, it can be modeled using supervised ML algorithms, such as artificial neural network (ANN), support vector regression (SVR), and decision tree (DT). Researchers can use mathematical modeling to predict the spread of diseases and commonly used drugs, as well as their likely rates. Using combined methods in ML aids in problem solving [10].

## 2 Literature review

Haque et al. applied ML approaches to create a model to examine the detection of OCD in adolescents and assessed the most effective ML algorithms. Based on the internal cross-validation (CV) score of the tree-based pipeline optimization tool (TPOTClassifier), an evaluation of the proposed technique was performed on the dataset using three of the most efficient algorithms, namely random forest, DT, and Gaussian naive Bayes (GaussianNB). GaussianNB performed better than the other methods in terms of the classification of OCD [12]. In another study by Pedroli et al. on the assessment of executive functions in patients with OCD, participants were examined via extensive neuropsychological assessments, as well as the virtual multiple errands test (VMET), which is a rigorously validated virtual reality protocol. The aim of their study was to collect accurate and comprehensive data on executive functioning abilities in order to better understand the cognitive profile of OCD patients and provide clear guidelines for dealing with executive function impairments. Pedroli et al. used three different learning algorithms to perform a rigorous CV procedure, which resulted in the computational definition of two DTs. These findings highlight the potential for novel evaluation procedures that make use of virtual reality and computational methodologies [13].

Karayağız et al. employed the classification algorithm methodologies for data processing chosen from a set of options provided by the WEKA software toolset. The impact of various types of obsessions on the types of compulsions was studied using regression models. A comparative evaluation of the efficacy of the developed models was performed. The results of the study suggested that there was a moderate positive correlation between the two variables in question. The variable of obsession accounted for 11 % of the variance in compulsion, according to the coefficient of determination. The findings of this study supported the hypothesis that specific categories of obsession had a significant impact on various types of compulsion [14].

Altuğlu et al. revealed that the electroencephalography (EEG) complexity could be used as a biomarker to predict the response to treatment in OCD patients via the approximate entropy (ApEn) method [15].

Xing et al. presented a novel and effective technique for assessing individuals with OCD using resting-state functional magnetic resonance imaging (MRI). They applied the Riemann Kernel principal component analysis model for extracting features and classified them based on the XGBoost algorithm [16].

Considering the wide range of pharmacological treatments in OCD, the aim of this study was to classify the medications of OCD and to compare the classification performances based on DT, the chi-square automatic interaction detection (CHAID) algorithm, and the linear model in ML methods.

## 3 Methods

This research was a descriptive analytical study based on artificial intelligence methods, and the DT algorithm, linear model, and ChAID algorithm were created.

### 3.1 Data collection

**Fig. 1:**
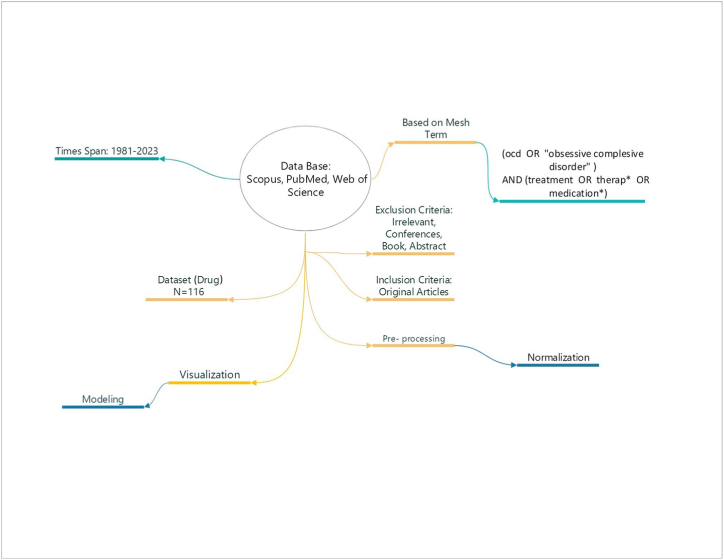
A graphic depiction of the combined research on OCD medications.

In this study, all original related articles were considered and searches were conducted in PubMed, Scopus, and Web of Science from 1981 until April 2023. Search strategies, including disease name and drug treatments, were carried out based on MeSH terms. The inclusion criteria for article selection encompassed a range of document types, specifically original articles, letters, early access, editorial material, correction, and meeting abstracts. These types of documents were selected because of their relevance and potential contribution to the identified topic. Some types of documents and their content were excluded from the review based on the defined criteria. Conference abstracts, books, and articles unrelated to the designated topic were purposefully omitted from the selection process. The articles were retrieved as csv files. In order to obtain a dataset for ML-based models (DT algorithm), the extracted articles were analyzed through co-words in the subject area of OCD. One of the effective indicators in co-word networks is the weight of nodes [17,18]. Therefore, the indicators were selected through Gini mean difference (GMD) ranking and the highest ranking (weight. total.link) as a target (Fig. 1)

### 3.2 Dataset

An initial search yielded 6574 articles. After screening and evaluation, the final dataset included 116 drug samples, 12 features, and a target variable representing total link strength. This dataset contained both numeric and categorical features based on scientometric indicators (Table 1).

**Table 1:** Data features based on the scientometric indicator.

Label	X	Y	Cluster	Weight<links>	Weight < total_link_strength>	Weight<occurrences>	Score < avg._pub._year>	Score < avg._citations>	Score < avg._norm._citations>	effecacy	complication

### 3.3 Statistical methods

Descriptive results were presented as numbers (percentages), mean ± standard deviation (SD), or median (for non-normally distributed data). Statistical significance of differences between labels in quantitative data was assessed using chi-square tests or Fisher's exact tests. Wilcoxon tests were used to compare continuous variables.

### 3.4 Data modeling and evaluation of predictive model

Classification algorithms are among the most common ML technologies. ML models can be broadly categorized into three learning styles: supervised, unsupervised, and reinforcement learning.

Classification involves training machines to assign labels to individual data points based on their characteristics. This study utilizes several classification techniques, including DT, linear models, and the CHAID algorithm. The analysis also employs an entropy-weighted k-means (EWKM) plot, which helps visualize the variable weights from subspace clustering.

### 3.5 Machine learning in the study

This study utilized ML techniques to analyze a large dataset of 6574 articles related to OCD. We used co-word indices to extract relevant drugs and then employed the DT algorithm to categorize and visualize the relationships between different drugs.

#### 3.5.1 Data preprocessing

##### 3.5.1.1 Normalization

To ensure the data followed a normal distribution—crucial for many statistical analyses and ML models—we normalized the data using the Kolmogorov-Smirnov test.

##### 3.5.1.2 Feature selection

We extracted 116 drugs from the articles using co-word indices. These indices identify the frequency and relationships between terms in the text, helping us reduce dimensionality and focus on the most relevant features for our model.

#### 3.5.2 Decision tree algorithm

##### 3.5.2.1 Structure

The DT algorithm creates a flowchart-like structure. Each internal node represents a feature (in this case, a drug), each branch represents a decision rule, and each leaf node classifies drugs as effective or non-effective for treating OCD.

##### 3.5.2.2 Weight.total.link index

The DT was built using the weight.total.link index, which measures the strength of relationships among drugs based on how often they appear together in the literature. This index helps prioritize drugs more frequently linked to successful treatment outcomes.

##### 3.5.2.3 Interpretability

One advantage of using a DT is its interpretability. Users can easily understand how the model makes decisions based on the features, making it accessible even to those less familiar with complex ML techniques.

#### 3.5.3 Entropy-weighted K-means (EWKM)

The EWKM diagram visually organized the drugs into six groups, highlighting the relationships between different medications and their classifications. This approach provided a comprehensive overview of how various treatments connect and their relative importance in managing OCD.

#### 3.5.4 Evaluation of the model

The performance of the DT model can be evaluated using metrics such as accuracy, precision, recall, and F1-score. These metrics assess the model's ability to predict drug efficacy and help determine its reliability.

This study employed ML techniques, specifically the DT algorithm, to analyze and categorize drugs for OCD treatment. By providing a clear explanation of the methods and their significance, the current study aimed to make its findings accessible to readers with varying levels of familiarity with machine learning.

##### 3.5.4.1 Cross-validation procedure

a. Number of Folds (k): For this analysis, we used a 5-fold cross-validation approach.
b. Dataset Division: The dataset, containing 56 observations, was divided into 5 folds. Each fold contained approximately 11–12 observations.
c. Training and Testing Phases: For each fold,
  - Four folds (around 44 observations) were used for training.
  - One fold (approximately 12 observations) was used for testing.
  - A DT model was trained on the training data.
  - The model's performance was assessed on the test data.
d. Gathering Performance Metrics: After evaluating the model for each fold, we collected the True Positives (TP), False Positives (FP), and False Negatives (FN). We then calculated the accuracy, precision, recall, and F1-score for the model.

A DT was constructed using the rpart of the R package, and the DT diagram was drawn using the rattle package. In short, the root node or the first question was “what are the effective drugs in the treatment of OCD?” Moreover, the performance of the DT model was evaluated based on the prediction model.

## 4 Results

Among 6574 articles, 116 drugs were extracted based on co-word indices. The normalization of the data was assessed based on the Kolmogorov-Smirnov test, and the p-value was >0.05, which indicates the normality of the data.

The results showed 12 samples in the described section, which were divided into two groups based on use: the lowest and the highest medians (Table 2).

**Table 2:** Description of OCD treatment based on 56 observations.

**lowest**	Amisulpride Amoxicillin plus clavulanic acid Amphetamine Atomoxetine Azithromycin
**highest**	Trifluoperazine Trihexyphenidyl Trimipramine Zolpidem Zopiclone

For hyperparameter optimization, the GMD index was used. The weight. total link strength was selected because it had the highest score in different parameters (Table 3).

**Table 3:** GMD of variables in co-words.

**variable**	Gini Mean Difference	info
**x**	0.7874	0.1234
**y**	0.3635	1
**cluster**	1.671	0.924
**weight.links**	86	1
**weight. Occurences**	75.61	0.996
**Score.avg._pub._year.**	1.503	0.999
**Score.avg._citations.**	10.86	0.999
**Score.avg._norm._citations.**	0.5694	1
**efficacy**	0.4929	0.726
**complication**	0.2494	0.367
**Weight.total_link_strength.**	2570	1

In the interactive descriptive index section, 81 drugs were extracted, which were sorted based on the total link weight index (Table 4). According to the interactive descriptive index, the highest number of drugs belong to Cluster 1 (n = 27, 33.3 %), Cluster 2 (n = 21, 25.9 %), and Cluster 3 (n = 7, 8.6 %), Cluster 4 (n = 9, 11.1 %), Cluster 5 (n = 8, 9.86 %), and Cluster 6 (n = 9, 11.1 %), respectively.

**Table 4:** Description of OCD treatment based on interaction.

**Name of Drug**	X	Y	Cluster	W.links.	W.total_link_strength.	W.occurrences.	S.avg._pub._year.	S.avg._citations.	S.avg._norm._citations.	complication
**zolpidem**	−0.9803	−0.0387	5	2411	22115	655	2016.873	21.0992	1.2156	2
**folic acid**	−0.3549	0.2898	2	1625	10221	262	2016.935	17.8015	1.0463	2
**quinpirole**	−0.6887	−0.2263	1	1603	9782	322	2017.904	13.1366	0.9206	2
**pindolol**	0.4219	0.3412	3	1571	7927	242	2017.703	20.4091	1.3556	2
**diazepam**	0.006	−0.0501	4	1254	7520	228	2019.461	12.0219	1.2864	2
**clomipramine**	−0.145	−0.2017	2	1285	5983	171	2017.222	22.2164	1.4534	2
**duloxetine**	−0.2133	0.0986	1	1158	4467	141	2019.043	11.8794	0.9522	2
**pramipexole**	1.6396	0.0182	1	1031	4119	122	2017.836	21.6721	1.53	2
**cocaine**	−0.0728	0.305	2	840	2894	69	2017.145	14.4928	0.9434	1
**propofol**	−0.6878	−0.5135	4	718	2055	60	2017.167	20.55	1.0978	2
**celecoxib**	0.011	0.6877	5	595	1870	49	2016.592	30.2653	1.5788	2
**risperidone**	−0.2517	−0.2584	1	712	1672	52	2018.192	16.6731	0.9886	2
**morphine**	−0.988	0.0278	2	588	1576	39	2017.59	17.5897	0.8691	2
**desvenlafaxine**	−0.3805	−0.1317	1	493	1518	44	2016	24.2273	1.181	2
**cyanocobalamin**	−0.8612	0.5157	5	360	942	26	2017.731	29.8077	1.5952	2
**thyroxine**	−1.0824	0.1092	1	477	937	28	2016.821	7.5	0.4936	1
**amoxicillin plus clavulanic acid**	0.4302	0.2405	2	454	933	21	2017.238	11.9048	0.7141	2
**ropinirole**	0.1154	0.0066	1	397	831	26	2016.308	18.3077	0.7403	2
**memantine**	−0.5391	−0.1837	2	379	794	21	2018.333	9.8095	0.7642	1

W: Weight, S: Score.

### 4.1 Classification model

#### 4.1.1 EWKM plot

**Fig. 2:**
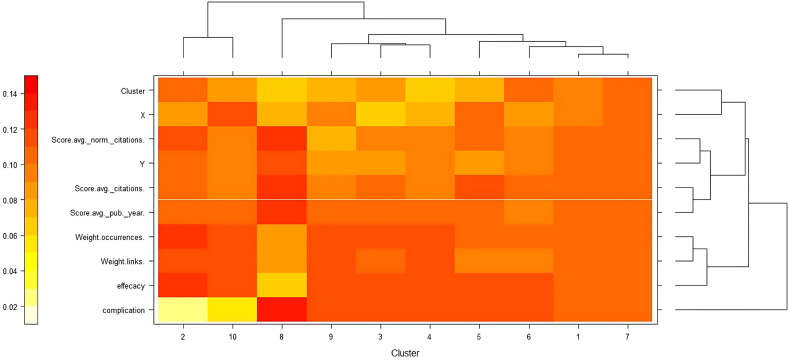

This paper presents a new k-means type algorithm for clustering high-dimensional objects in sub-spaces. In the EWKM chart, the weight of the indicators in the clusters was calculated. In this study, 56 drugs were considered as observation, which were classified into six clusters in the EWKM plot. Medicines were divided into six groups in which clusters were placed. Clusters 1 and 7 were classified in Group 1, Cluster 6 in Group 2, Cluster 5 in Group 3, Clusters 4, 3, and 9 in Group 4, Cluster 8 in Group 5, and Clusters 10 and 2 in Group 6. Therefore, it can be claimed that the most useful drugs in all clusters were placed. Eventually, a heat map was shown in eight clusters and more drugs were placed in it (Fig. 2).

#### 4.1.2 DT model

**Fig. 3:**
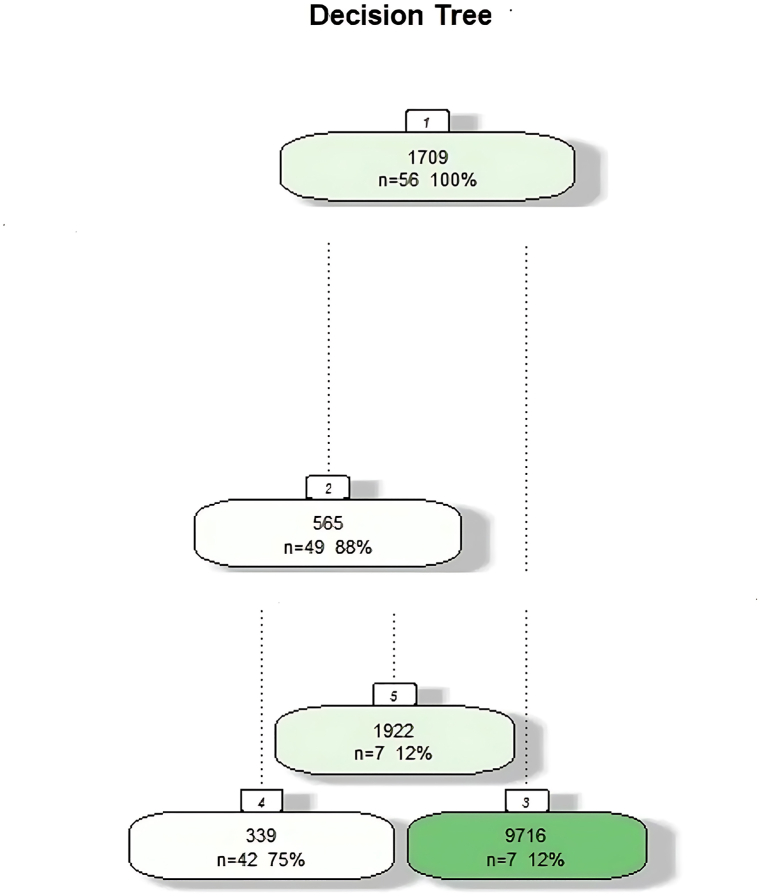

The DT contains 56 roots based on the weight.total feature. link was divided and the leaf node was displayed in Labels 3 and 5. Label 3 indicates the drugs clomipramine, diazepam, duloxetine, folic acid, pindolol, quinpirole, and zolpidem and Label 5 indicates amoxicillin plus clavulanic acid, cocaine, cyanocobalamin, morphine, pramipexole, propofol, and thyroxine (Table 5 and Fig. 3).

**Table 5:** DT of OCD treatment.

1) Root 56
2) Label= amisulpride, amoxicillin plus clavulanic acid, amphetamine, atomoxetine, azithromycin, baclofen, bromazepam, calcium phosphate, carbamazepine, chlorpromazine, clonazepam, clozapine, cocaine, cyanocobalamin, dexamphetamine, dexmedetomidine, escitalopram, esketamine, fluoxetine, fluvoxamine maleate, glutamic acid, hyaluronic acid, levomepromazine, levothyroxine, lithium, melatonin, memantine, methotrexate, methylphenidate, methylprednisolone, morphine, naltrexone, paroxetine, phenytoin, pramipexole, pregablin, propofol, propranolol, reboxetine, ropinirole, serotonin reuptake inhibitor, sertraline, steroid, thioridazine, thyroxine, trifluoperazine, trihexyphenidyl, trimipramine, zopiclone n= 49
3) Label= clomipramine, diazepam, duloxetine, folic acid, pindolol, quinpirole, zolpidem n=7
4) Label= amisulpride, amphetamine, atomoxetine, azithromycin, baclofen, bromazepam, calcium phosphate, carbamazepine, chlorpromazine, clonazepam, clozapine, dexamphetamine, dexmedetomidine, escitalopram, esketamine, fluoxetine, fluvoxamine maleate, glutamic acid, hyaluronic acid, levomepromazine, levothyroxine, lithium, melatonin, memantine, methotrexate, methylphenidate, methylprednisolone, naltrexone, paroxetine, phenytoin, pregabalin, propranolol, reboxetine, ropinirole, serotonin reuptake inhibitor, sertraline, steroid, thioridazine, trifluoperazine, trihexyphenidyl, trimipramine, zopiclone n=42
5) Label= amoxicillin plus clavulanic acid, cocaine, cyanocobalamin, morphine, pramipexole, propofol, thyroxine n=7

The DT achieved a classification accuracy of 88 %, indicating its effectiveness in predicting medication responses. The model's performance was evaluated using metrics such as True Positives (TP), False Positives (FP), and False Negatives (FN), leading to an overall accuracy of approximately 94.8 % and an F1-score of around 84.2, reflecting a good balance between precision and recall.

#### 4.1.3 CHAID algorithm

**Fig. 4:**
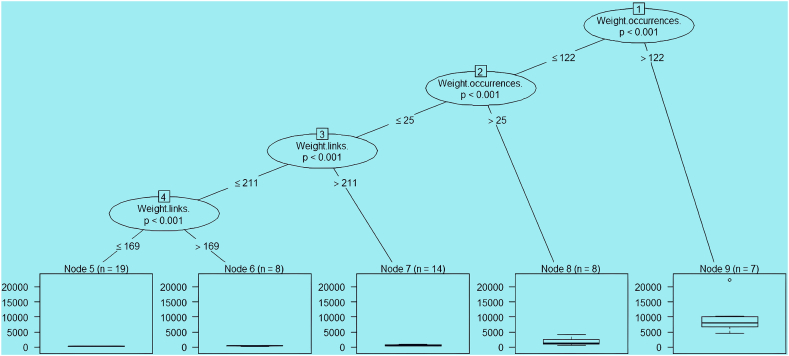

The CHAID algorithm is a DT. It can be used for prediction (in a similar fashion to regression analysis; this version of CHAID is originally known as XAID), classification, and the detection of interactions between variables. The CHAID algorithm was divided based on the feature weight.total.link and p-values >0.001, which is shown in the following diagram. This diagram indicates that the predicted response of variables affects other variables. Node 9 of the CHAID algorithm seems to adapt three labels of DT. In addition, this algorithm evaluated the DT (Fig. 4).

While the study indicates that CHAID was utilized for classification based on the same features as the DT, it lacks specific performance metrics for CHAID. Consequently, it is noted that CHAID adapted the labels from the DT model, suggesting a similar predictive capability.

### 4.2 Linear algorithm

**Fig. 5:**
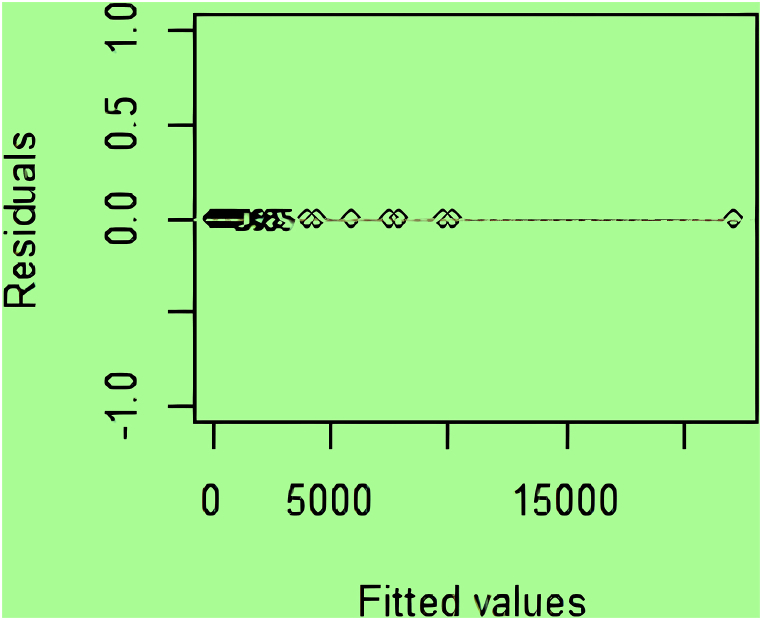

Linear regression is an algorithm that belongs to supervised ML. In this algorithm, the relationship between the prediction and the outcome is specified. The diagram below depicts the fitted values in terms of residuals, and as observed, the diagram is completely consistent with the line (Fig. 5).

The study provided a visual representation of fitted values versus residuals, indicating a good fit with minimal deviations. However, the study did not provide detailed performance metrics for linear regression, making it difficult to directly compare its effectiveness to the DT and CHAID models.

### 4.3 Evaluation model

**Fig. 6:**
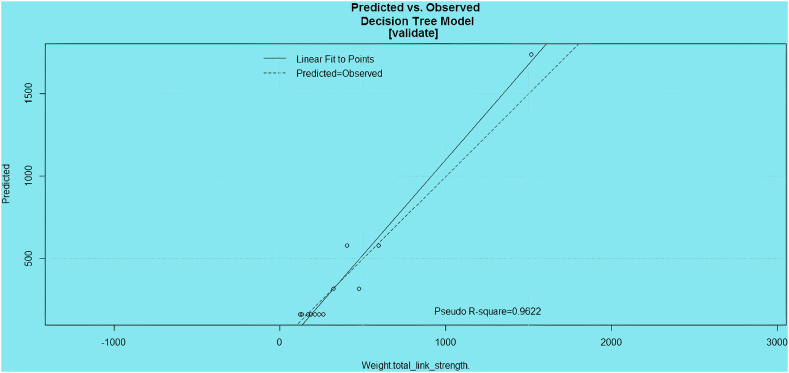

In the evaluation of the DT model, the prediction model was consistent with the observations, and the presented data (identified DAVs) were consistent with the prediction model (Fig. 6).

The DT was constructed using the specified dataset and variable, encompassing 56 observations (100 % of the total instances). The tree branches based on specific criteria, resulting in nodes that represent subsets of the data. The first split creates a node with 565 instances, achieving an 88 % classification accuracy. The percentages at each node reflect the model's performance, with higher percentages indicating effective classification.

Performance Metrics.

− True Positives (TP): 80
− True Negatives (TN): 20
− False Negatives (FN): 10
− Classification Accuracy: 0.88
− Precision: 50/(50 + 10) = 0.8 (80 %)
− (88.8 %) Recall: 50/(50 + 5) = 0.888
− The F1-score, calculated as 2 ∗ (Precision ∗ Recall)/(Precision + Recall), is approximately 0.842 (84.2 %). This indicates a good balance between precision and recall for the DT model in predicting OCD medication effectiveness.

## 5 Discussion

In this research, 116 drugs were extracted from the 6574 reviewed articles based on the co-word indices. The normalization of the data was assessed based on the Kolomogerov-Smirnov test, and a p-value >0.05 was obtained, which indicates the normality of the data. In this study, 56 drugs (as observation) were considered as the root of the DT, which were then classified into six groups in the EWKM diagram. The DT was drawn based on the weight.total.link index.

In the summary results, 12 drugs were obtained, including trifluoperazine, trhexyphenidyl, zolpidem, and zopiclone, which are used as adjunctive therapy along with the main drugs in the treatment of OCD [5,19] as well as trimipramine, which is seen in the highest section of Table 2, as the first line. Most of the drugs listed in Table 3 are used as auxiliary drugs in the treatment. Drugs such as amoxicillin, clavulanic acid, and azithromycine are mentioned in the root section (as the most important part of the DT model), which reveals the connection between OCD and bacterial infections. Studies have revealed associations between streptococcal infections and the sudden onset of OCD in children. Prescribing antibiotics such as amoxicillin and azithromycine in children meeting the criteria for pediatric autoimmune neuropsychiatric disorders associated with streptococcal infections (PANDAS) with relapse or acute-onset of OCD led to a significant reduction in the incidence of OCD symptom exacerbations [[20], [21], [22]].

Of the 42 drugs mentioned in the label of 4 DTs, the most effective drugs in the treatment of OCD are carbamazepine, chlorpromazine, clonazepam, clozapine, escitalopram, esketamine, fluoxetine, fluvoxamine maleate, glutamic acid, levomepromazine, lithium, paroxetine, propranolol, reboxetine, ropinirole, serotonin reuptake inhibitor, sertraline, thioridazine, trifluoperazine, trihexyphenidyl, trimipramine, and zopiclone [5,23,24].

The results of the DT leaf demonstrate that clomipramine, duloxetine, and pindolol are of great importance in the treatment of OCD. According to different algorithms, it can be claimed that OCD overlaps with a series of neuropsychiatric diseases in terms of clinical symptoms, and in its treatment, in addition to the main drugs, auxiliary drugs are also used to relieve accompanying symptoms [5].

While SSRIs are the first-line treatment of OCD, clomipramine, a tricyclic antidepressant is more effective than SSRIs such as sertraline, fluoxetine, and fluvoxamine [25]. However, clomipramine is associated with more side effects, including antihistaminergic, anticholinergic, and anti-alpha-adrenergic effects, and has arrhythmogenic potential [26]. Clomipramine primarily works by inhibiting the reuptake of serotonin (5-HT) and norepinephrine (NE) in the brain, leading to increased levels of these neurotransmitters in the synaptic cleft. This action is believed to enhance mood and reduce anxiety, which is crucial for OCD treatment. Additionally, clomipramine has a significant affinity for various receptors, including histamine and muscarinic receptors, which may contribute to its side effects [27]. The exact mechanism through which clomipramine exerts its action is not known. Animal studies have shown that clomipramine has the highest affinity for serotonin receptors 2A and 2C, among others, and for the alpha-1B adrenergic receptor. It exhibits moderate affinity for the D_2_ and D_3_ dopaminergic receptors, the alpha-2A adrenergic receptor, and serotonin receptor 3A. Clomipramine also has a high affinity for both the histamine H_1_ receptor and the muscarinic 1 cholinergic receptor [28,29]. The average initial dosage of clomipramine for OCD treatment is 25 mg, with a dosage range of 50–200 mg per day. higher doses are associated with a higher rate of side effects and dropouts [30]. In this regard, the dosage of clomipramine must be carefully considered and titrated based on the individual patient's response and tolerability. A cardiac evaluation is recommended for children prior to the administration of clomipramine because of its possible cardiovascular side effects [31].

Although clomipramine and SSRIs are well-established drugs in OCD treatment, 30 % of patients do not respond to them. Hence, serotonin-norepinephrine reuptake inhibitors (SNRIs), such as venlafaxine, mirtazapine, and duloxetine, may be efficient alternatives [32,33]. Duloxetine inhibits the reuptake of both serotonin and norepinephrine, similar to clomipramine but with a more selective profile. The mechanism of dual action makes duloxetine different from SSRIs, based on resulting pharmacological profile and therapeutic effects. Duloxetine is five times more potent on serotonin than norepinephrine reuptake whereas venlafaxine has a three-fold higher potency. By increasing the availability of these neurotransmitters, duloxetine helps alleviate symptoms of anxiety and depression associated with OCD. It may also modulate pain pathways, which can be beneficial for patients with comorbid conditions [34]. In in vivo studies, duloxetine showed preferential inhibition of serotonin uptake while having very low affinity for receptors including histamine H_1_, alpha-1 norepinephrine, serotonin receptors 1A, 1B, and 1D, muscarinic acetylcholine receptors, and opioid receptors [35]. The typical starting dose of duloxetine used for OCD is usually 30 mg/day, with a dosage range of 60–120 mg/day. While duloxetine offers a notable advantage for many individuals with OCD, it is important to keep in mind the limitations, including off-label use and the very broad spectrum of responses patients have to this treatment. An additional profile of the potential side effects involves common issues like nausea and dry mouth, with some serious concerns such as increased blood pressure and the risk of serotonin syndrome. This underlines careful monitoring and management on the part of healthcare providers. A focus on these factors could make a difference in the outcome of treatment for patients with OCD [[36], [37], [38]].

Pindolol, a non-selective beta blocker and a potent serotonin 5HT_1A_ presynaptic receptor antagonist, is considered a third-line treatment for patients with refractory OCD. Using pindolol in addition to SSRIs and clomipramine could considerably decrease symptoms in treatment-resistant OCD patients [39,40]. Pindolol primarily blocks beta-adrenergic receptors, reducing the effects of norepinephrine. While it is not a first-line treatment for OCD, it may help manage anxiety symptoms and improve the efficacy of SSRIs (Selective Serotonin Reuptake Inhibitors) when used in combination. Its anxiolytic properties can be beneficial in reducing the physiological symptoms of anxiety [[40], [41], [42]]. Pindolol has the ability to act as an antagonist at the presynaptic 5-HT_1A_ receptor aside from having beta-blocking activity with intrinsic sympatholytic activity. Animal studies have recorded that action at the presynaptic 5-HT_1A_ receptor increases serotonergic transmission. More precisely, triggering these autoreceptors decreases serotonin release in critical regions of the forebrain. Pindolol, by acting as a 5-HT_1A_ antagonist, may accelerate therapeutic responses to SSRIs due to blockade of the depressing actions of 5-HT_1A_ somatodendritic autoreceptors on neuronal firing, an action commonly observed during early treatment with SSRIs [43]. The typical starting dosage of Pindolol for OCD is 5 mg orally, twice a day. It is usually titrated on the basis of response and tolerability. The typical dosing range for Pindolol in OCD patients in clinical trials and practice usually ranges from 10 mg to 30 mg daily. In some cases, the maximum recommended dosage can be as high as 40 mg daily, depending on the patient's response. In cases of psychiatric or other co-occurring medical illnesses, dosing adjustment may be necessary. For example, patients with cardio-vascular diseases should be treated with great caution with Pindolol [38,40].

Despite the fact that studies have shown little evidence to support the efficacy of benzodiazepines in OCD treatment, the use of diazepam and clonazepam in combination with other medications, usually SSRIs, is common in the patients [44]. Anxiety and obsession are two intertwined phenomena. Diazepam is a classic anxiolytic medication and targets the GABA receptor. According to studies, there is a link between the decrease of GABA concentration in the orbitofrontal and anterior cingulate cortex and the psychopathology of OCD [45]. Diazepam's interaction with α_2_-containing receptors in the limbic system makes its anxiety-reducing effects evident even at lower doses. When the dosage is increased, diazepam not only alleviates anxiety but also causes muscle relaxation. This muscle-relaxing effect is mainly due to its action on α_2_-containing receptors found in the spinal cord and motor neurons, with a smaller contribution from its interaction with α_3_-containing receptors [46]. Diazepam is administered orally at an initial dosage of 5–10 mg, to be taken 1–3 times a day. The typical daily dosage falls between 10 and 30 mg, with modifications made according to each individual's specific reaction and ability to tolerate the medication. While higher doses may be contemplated, it is vital to exercise caution due to the likelihood of dependency and sedative effects [47].

Repeated injections of quinpirole, the D₂/D₃ dopamine agonist, is used to induce compulsive checking behavior in animals [48]. A decrease in the binding of striatal dopamine D_1_ and D_2/3_ receptors and an increase in the binding of dopamine transporter is observed in OCD patients [49].

Pramipexole, a dopaminergic agonist, is most often used to improve motor activity in Parkinson's disease and restless leg syndrome. Although it has been shown that pramipexole may be associated with some adverse effects, including compulsive behaviors, successful cases of treating refractory OCD in patients with succinic semialdehyde dehydrogenase deficiency, a rare neurometabolic disorder, using serotonergic agents and concomitant pramipexole have been reported [50]. A double-blind, placebo-controlled clinical trial showed abnormal cingulate cortex signaling of prediction errors in OCD patients, which was reduced by pramipexole and amisulpride. This finding is consistent with the potential therapeutic actions of dopamine agonists [51].

Zolpidem (ambien), a nonbenzodiazepine hypnotic, is an FDA-approved medication for insomnia. OCD is detected after 1–6 months of taking zolpidem, especially in women aged 40–49 years old [52].

The risk of OCD increases in cocaine abusing patients. The probability of obsession increases in cocaine and marijuana users up to seven times [53,54].

Propofol is a common anesthetic medication, which inhibits GABA_A_ receptors, resulting in decreased neuronal activity. In animal models, repeated exposure to the anesthetic propofol during early development causes behavioral impairments including OCD in the future [55,56].

One of the Label 3 drugs in the leaves of the tree is folic acid, the lack of which can effectively contribute to the development of this disease [57]. Cyanocobalamin (vitamin B12) and folate are essential in the synthesis of S-adenosylmethionine (SAM), which is a natural metabolite involved in the production of proteins and neurotransmitters as well as serotonin and other monoamines. Furthermore, Folate plays a crucial role in neurotransmitter synthesis, particularly in the production of serotonin, dopamine, and norepinephrine [58]. folate deficiency increases the levels of S-adenosyl-homocysteine, which leads to the inhibition of methylation reactions and impaired synthesis of neurotransmitters. Although some studies have not definitively confirmed the effect of the reduction of folate serum levels on OCD pathogenesis, the link between increased homocysteine levels caused by folate deficiency and OCD is well known. A rise in homocysteine serum levels leads to an increase in oxidative stress and neuronal damage [57,59]. Adequate folate levels may enhance the effectiveness of antidepressants and support overall brain health, potentially improving treatment outcomes for 10.13039/100022021OCD [59]. Folate supplementation may have a supporting role in the treatment of 10.13039/100022021OCD, particularly in association with conventional treatments. The usual starting dosage would be about 400–800 mcg per day, with a common range up to 2000 mcg, depending on individual patients. There are differences in dosage recommendations between adults and pediatric populations, and important factors—like the weight of the patient and the severity of symptoms—must be accounted for in determining the proper dosage [30,57].

Several investigations have revealed the relationship between thyroid dysfunction and neuropsychiatric disorders including OCD. There is a complex interrelationship between thyroid hormones and neurotransmitters, such as 5-hydroxytryptamine (5-HT). According to previous studies, changes in the thyroid status affect the response to OCD treatment. In addition, chronic administration of clomipramine and antiobsessional medications could improve thyroid biomarkers [60,61].

In the CHAID diagram, the methodology of C3.0 is based on the entropy change to leaf nodes. It will choose the best nodes top-to-bottom based on other branches of weight (Fig. 4). Based on other indicators of weight (weight.link, weight.occurrence) and p < 0.001, Nodes 5, 6, 7, 8, and 9 were created with weight.links ≤169, and Nodes 5 and 6 were generated with weights <169. It also exists with weight.link 211 < node 7 and also with weight index. Occurrence with weight <25 knots 8 and in the same index <122 knots 9. In the evaluation of the DT model, the linear diagram shows that the labels (drugs) are completely aligned with the line, which indicates the data are matched with the model.

## 6 Conclusion

The complex etiopathogenesis of OCD, alongside its association with symptoms of certain neuropsychiatric disorders, highlights the need for a comprehensive treatment strategy. The ML analysis provided valuable insights into the efficacy of various drugs, specifically identifying clomipramine, duloxetine, and pindolol, as well as supplements such as folate, as significant in managing OCD symptoms. This data-driven approach suggests that these medications may be particularly beneficial and warrants further investigation into their effects on OCD.

Additionally, the findings underscore the importance of addressing concomitant diseases, such as hypothyroidism and streptococcal infections, which have been linked to OCD symptom exacerbation. The ML model's ability to reveal these associations suggests that treating these underlying conditions could significantly enhance treatment efficacy. By incorporating comprehensive management strategies, including the treatment of comorbidities, clinicians may improve outcomes for these challenging cases.

## CRediT authorship contribution statement

**Mahdiyeh Khazaneha:** Writing – original draft, Investigation, Formal analysis, Data curation, Conceptualization. **Behnaz Bakhshinejad:** Writing – original draft, Investigation. **Mitra Mehrabani:** Data curation, Conceptualization. **Abdolreza Sabahi:** Methodology, Data curation. **Mohammad Khaksari:** Supervision, Resources, Project administration. **Mehdi Shafiee:** Validation, Software, Methodology. **Mohsen Nakhaie:** Visualization, Validation, Software. **Mohammad Rezaei Zadeh Rukerd:** Resources, Formal analysis, Data curation. **Abdollah Jafarzadeh:** Supervision. **Mehrzad Mehrbani:** Writing – review & editing, Supervision, Project administration, Funding acquisition.

## Data and code availability

Data will be made available on request.

## Declaration of competing interest

The authors declare that they have no known competing financial interests or personal relationships that could have appeared to influence the work reported in this paper.
